# Powerful Plant Antioxidants: A New Biosustainable Approach to the Production of Rosmarinic Acid

**DOI:** 10.3390/antiox9121273

**Published:** 2020-12-14

**Authors:** Abbas Khojasteh, Mohammad Hossein Mirjalili, Miguel Angel Alcalde, Rosa M. Cusido, Regine Eibl, Javier Palazon

**Affiliations:** 1 Laboratori de Fisiologia Vegetal, Facultat de Farmacia, Universitat de Barcelona, Av. Joan XXIII sn, 08028 Barcelona, Spain; abbaskhojasteh@aol.com (A.K.); miguel.psr.94@gmail.com (M.A.A.); rcusido@ub.edu (R.M.C.); 2 Department of Agriculture, Medicinal Plants and Drugs Research Institute, Shahid Beheshti University, 1983969411 Tehran, Iran; m-mirjalili@sbu.ac.ir; 3 Campus Grüental, Institute of Biotechnology, Biotechnological Engineering and Cell Cultivation Techniques, Zurich University of Applied Sciences, CH-8820 Wädenswill, Switzerland; eibs@zhaw.ch

**Keywords:** rosmarinic acid, savory, lamiaceae, oxidative stress, phenolic compounds, cell cultures

## Abstract

Modern lifestyle factors, such as physical inactivity, obesity, smoking, and exposure to environmental pollution, induce excessive generation of free radicals and reactive oxygen species (ROS) in the body. These by-products of oxygen metabolism play a key role in the development of various human diseases such as cancer, diabetes, heart failure, brain damage, muscle problems, premature aging, eye injuries, and a weakened immune system. Synthetic and natural antioxidants, which act as free radical scavengers, are widely used in the food and beverage industries. The toxicity and carcinogenic effects of some synthetic antioxidants have generated interest in natural alternatives, especially plant-derived polyphenols (e.g., phenolic acids, flavonoids, stilbenes, tannins, coumarins, lignins, lignans, quinines, curcuminoids, chalcones, and essential oil terpenoids). This review focuses on the well-known phenolic antioxidant rosmarinic acid (RA), an ester of caffeic acid and (*R*)-(+)-3-(3,4-dihydroxyphenyl) lactic acid, describing its wide distribution in thirty-nine plant families and the potential productivity of plant sources. A botanical and phytochemical description is provided of a new rich source of RA, *Satureja khuzistanica* Jamzad (Lamiaceae). Recently reported approaches to the biotechnological production of RA are summarized, highlighting the establishment of cell suspension cultures of *S. khuzistanica* as an RA chemical biofactory.

## 1. Introduction

Unhealthy modern lifestyles, marked by physical inactivity, obesity-inducing diets, and exposure to air pollution and other environmental and chemical stresses, are responsible for a wide range of diseases worldwide caused by excessive production of free radicals during biological metabolism [1,2,3]. Free radicals such as reactive oxygen species (ROS) and nitrogen species are unstable molecules or atoms formed when an electron bond in a stable molecule is broken. As they contain unpaired electrons in their last electron layer, they have an enhanced propensity to undergo chemical reactions [4]. Free radicals are ubiquitous in the living environment as well as in the human body, where they can cause irreparable damage to the surrounding tissues by destroying cells and releasing toxic substances [1].

The most important free radical in the human body is oxygen. Superoxide (O_2_^●−^), hydroxyl (OH^●^), and peroxyl (ROO^●^) radicals, and nonradical species such as hydrogen peroxide (H_2_O_2_) and singlet oxygen (^1^O_2_) are common ROS generated from oxygen after an electron is taken up from surrounding molecules upon exposure to various environmental stresses, such as UV radiation, air pollution, and smoking [1,5,6]. ROS cause the destruction of cells, carbohydrates, lipids, proteins, and nucleic acids [7,8] and their activity in the human body weakens the immune system and leads to various diseases such as cancer, diabetes, heart failure, brain damage, muscle problems, premature aging, and eye injuries [9,10].

The main system for free radical scavenging and the regeneration of damaged cells in the body involves antioxidants, which curb the effect of free radical molecules [11]. Consumption of foods rich in antioxidants is therefore essential for human health, strengthening the immune system as well as prolonging life and delaying the aging process [12,13]. Antioxidants are generally classified into two groups based on their mode of action, which can either inhibit or prevent oxidation. The primary antioxidants, called chain-breaking antioxidative compounds, react directly with lipid radicals and convert them into relatively stable products by supplying a hydrogen atom (H^●^) [14,15]. The other group, known as secondary antioxidants, can reduce the rate of oxidation, mainly by binding metal ions, and are able to catalyze oxidation by oxygen scavenging, absorbing UV radiation, and inhibiting enzymes [15]. Antioxidant enzymes (e.g., catalase, glutathione peroxidases, ascorbate peroxidase, and superoxide dismutase), vitamins (e.g., vitamin C, vitamin E, and β-carotene), extracellular proteins (e.g., albumin, transferrin, lactoferrin, haptoglobin, and ceruloplasmin), and other cellular compounds (e.g., quinones, glutathione, uric acid, and bilirubin) form the antioxidant defense system in the human body [6,16]. 

In the food industries, antioxidants are divided into synthetic and natural. Butylated hydroxyanisole (BHA), butylated hydroxytoluene (BHT), propyl gallate (PG), and tertbutylhydroquinone (TBHQ) are well-known synthetic antioxidants widely used in food products and beverages. However, their use is increasingly limited due to toxicity and carcinogenic effects [17,18]. The growing preference among consumers for food products free of any synthetic additives has stimulated an interest in natural antioxidants in the food industries. These plant-derived compounds, both primary and secondary metabolites, can exert antioxidative activity in a food model system [19,20]. 

Primary metabolites such as nucleic acids, amino acids, proteins, carbohydrates, antioxidative enzymes, and fatty acids are essential for plant growth and survival [21]. Produced during photosynthesis, they participate in the production of cellular compounds. In addition, plants synthesize a wide range of chemical compounds known as secondary metabolites (SMs) during their interactions with the environment, especially in response to biotic and abiotic stresses [22]. Based on their biosynthetic pathways and chemical structure, SMs are classified into different types (e.g. terpenoids, alkaloids, phenolics, flavonoids, and steroids), and are widely used in the pharmaceutical, food, cosmetic, and agrochemical industries [23,24]. The most important SMs are polyphenols, also called phenolic compounds, which are natural plant antioxidants. To date, approximately ten thousand phenolic compounds have been identified and isolated from plant taxa [25]. Due to their antimicrobial and antioxidant activity [26,27], plant-derived phenolic compound production from plant raw materials and by-products has attracted considerable attention over the last decades [28,29]. 

The sensitivity of medicinal plants to environmental changes, their low contents of valuable SMs, and the costly extraction of these compounds from raw materials, questions the feasibility of biologically active compound production using plants [30]. Alternatively, the biotechnological production of SMs through in vitro plant cell, tissue, and organ culture is an efficient and faster method that guarantees stable and controlled conditions. These in vitro techniques have been widely used for the production of several SMs, notably taxol [31,32,33], podophyllotoxin [34,35,36], withanolides [37], centellosides [38,39], and rosmarinic acid (RA) [40,41,42,43], as well as for mass propagation [44], in vitro cloning [45,46,47], and polyploidization [48,49]. Many RA-producing biotechnological platforms have been established, based on shoots [50,51,52], callus [53], cells [41,42,54], and hairy root cultures [55,56,57,58] of numerous species of the Boraginaceae, Anthocerotaceae, and Lamiaceae. 

In recent years, our research group has developed callus and cell suspension cultures of *Satureja khuzistanica* Jamzad (Lamiaceae), a rich source of RA [41,42,53,54], for further scaling up and commercial production of this valuable natural antioxidant. The present review is focused on the recent biotechnological and metabolic engineering achievements in the production of valuable natural antioxidants, with special emphasis on the use of *S. khuzistanica* in vitro cultures as a chemical RA factory.

## 2. Phenolic Compounds

Phenolic compounds, a large group of water-soluble plant SMs, comprise a variety of signal molecules, pigments, and flavorings that not only have protective roles against a variety of environmental stresses, pests, and diseases but are also instrumental in attracting pollinators due to their colors and sensory characteristics [20,59,60]. Together with antioxidative enzymes (e.g., catalase, peroxidase, guaiacol peroxidase, superoxide dismutase, and ascorbate peroxidase), polyphenols play an important protective role in scavenging free radicals during oxidative stress [60]. 

Phenolic compounds vary from low molecular weight monomers to high molecular weight polyphenols, and according to their number of phenolic hydroxyl (–OH) groups they are classified as *mono*-phenol (phenol), *di*-phenol (catechol, resorcinol, and hydroquinone), and *tri*-phenol (gallocatechol and phloroglucinol). 

As plant antioxidants, phenols can inhibit lipid peroxidation and exhibit various physiological activities [61]. Modeling the role of these natural antioxidants in defense mechanisms and adaptation processes has led to their use in the field of nutrition and human health. Widely used in the prevention and treatment of many chronic diseases such as cardiovascular conditions, diabetes, and cancer [62], polyphenols and phenol-rich plant extracts are also commercially used in the food and beverage industries as natural additives to control pathogenic and corrosive bacteria.

Horticultural fruit and vegetable crops, medicinal plants (e.g., mint, savory, rosemary, thyme, sage, basil, and oregano) and beverages are rich in phenolic compounds. By-products of food and agricultural industries, often generated in substantial quantities, are also potentially valuable sources of bioactive phenolic materials [59]. Phenolic acids, flavonoids, tannins, coumarins, lignans, lignins, naphtaquinones, anthraquinones, xanthones, and stilbenes [25,63] are well known phenolic compounds in the plant taxa (Table 1).

### Rosmarinic Acid 

**Figure 1:**
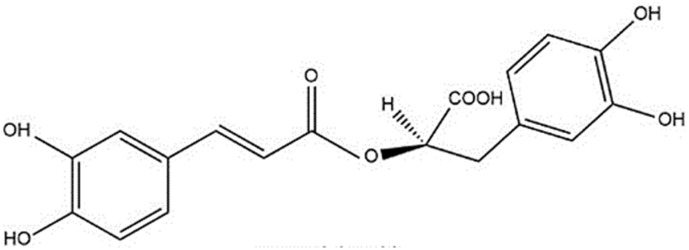
Chemical structure of rosmarinic acid.

Rosmarinic acid (RA) was first isolated from *Rosmarinus officinalis* L. (Lamiaceae) by Scarpati and Oriente in 1958. RA is an ester of caffeic acid and (*R*)-(+)-3-(3,4-dihydroxyphenyl) lactic acid (Figure 1), is prevalent in a wide range of plants, and a bioactive component of several medicinal plant species [64]. 

Caffeic acid is a structural unit of various types of secondary metabolites, ranging from the simplest monomers to multiple dense compounds and their oligomers. Trimers and tetramers of caffeic acid are reported to be therapeutic compounds with outstanding biological activities. Caffeic acid monomers are often found in the form of caffeic acid and 3-(3,4-dihydroxyphenyl) lactic acid [64,65]. Other monomeric derivatives include ferulic acid, isoferulic acid, and chlorogenic acid. The latter, in contrast with its high abundance in fruits, is rarely found in the Lamiaceae, where it has been replaced by RA [64].

RA is one of the most abundant caffeic acid dimers in plants and is known for its exceptional antioxidant activity [64] and its role in defense against pathogens and herbivores [66]. A number of RA derivatives have been identified in plants composed of one or two RA along with other aromatic groups, the most common being lithospermic acid and lithospermic acid B. 

Based on current knowledge, RA is distributed in 39 plant families, including hornworts, one of the earliest groups of land plants to evolve, as well as highly evolved monocotyledonous and eudicotyledonous species. In particular, RA has been isolated from many taxa of the Lamiaceae (*Ajuga*, *Agastache*, *Calamintha*, *Cedronella*, *Coleus*, *Collimsonia*, *Dracocephalum*, *Elsholtzia*, *Glechoma*, *Hornium*, *Lavandula*, *Lycopus*, *Melissa*, *Mentha, Micromeria*, *Monarda*, *Origanum*, *Perilla*, *Perovskia*, *Plectranthus*, *Salvia*, and *Satureja*) and Boraginaceae (*Cerinthe*, *Echium*, *Heliotropium*, *Lindefolia*, *Lithospermum*, *Nonea*, *Symphytum*, *Hydrophyllum*, *Nemophila*, and *Phacelia*), but not all the members of these families accumulate RA. This phenolic acid is also found in the other plant families, including *Chloranthus* spp. (Choranthaceae) and *Blechnum* spp. (Blechnaceae), as well as some orders of the monocotyledonous plants, and the rosids and asterids within the eudicotyledonous plants. The presence of RA in the marine hydrophilus angiosperms such as *Zostera marina* Linnaeus (eelgrass), *Z. noltii* Hornemann (dwarf eelgrass) has also been reported [67,68,69]. However, to date RA has not been reported in any of the gymnosperms [64,66].

**Figure 2:**
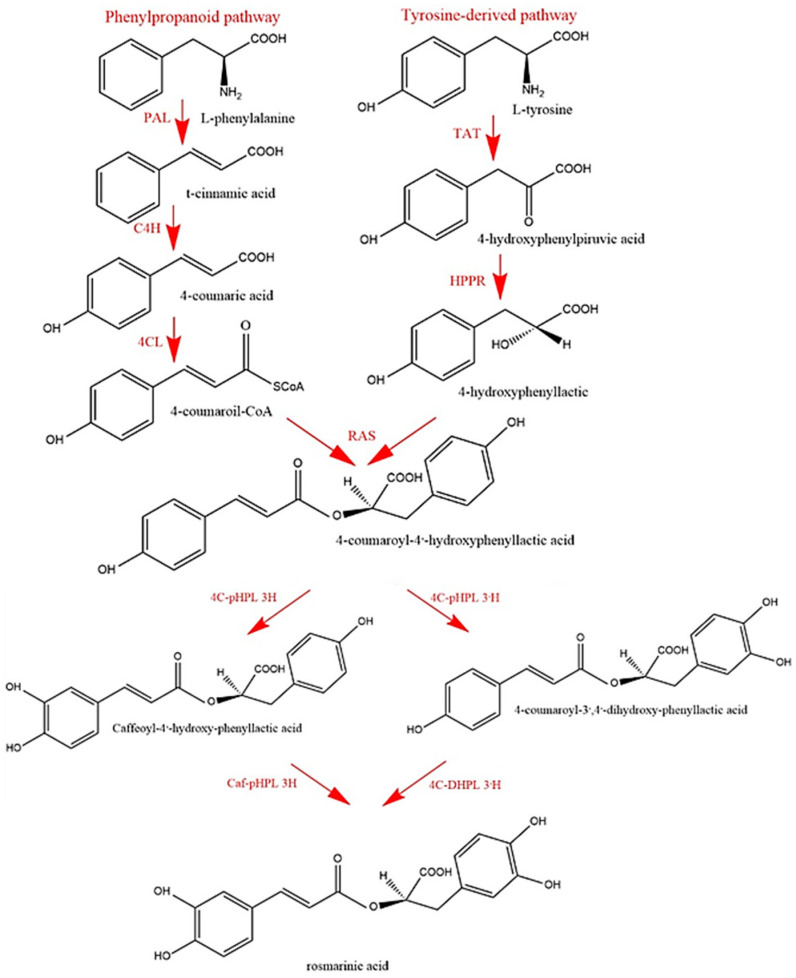
Scheme of the biosynthetic pathway of rosmarinic acid in *Coleus blumei*. PAL: phenylalanine ammonialyase, C4H: cinnamic acid 4-hydroxylase, 4CL: 4- coumarate:coenzyme A ligase, TAT: tyrosine aminotransferase, HPPR: hydroxyphenylpyruvate reductase, HPPD: hydroxyphenylpyruvate dioxygenase, RAS: rosmarinic acid synthase, 4C-pHPL 3/3′-H 4-coumaroyl-4′-hydroxyphenyllactate 3/3′-hydroxylase(s), Caf-pHPL 3H caffeoyl-4′-hydroxyphenyllactate 3-hydroxylase, 4C-DHPL 3H 4-coumaroyl-3,4-dihydroxyphenyllactate 3-hydroxylase (modified from Hücherig and Petersen, 2013 [74] ).

The biosynthetic pathway of RA has been comprehensively elucidated, and eight enzymes involved in the different steps have been characterized so far [70]. The initial precursors of RA are the aromatic amino acids L-phenylalanine and L-tyrosine, which are transformed to the intermediates 4-coumaroyl-CoA and 4-hydroxyphenyllactic acid (pHPL), respectively (Figure 2). 

Phenylalanine is converted to 4-coumaroyl-CoA by the enzymes of the phenylpropanoid pathway: phenylalanine ammonia-lyase (PAL), cinnamic acid 4-hydroxylase (C4H), and 4-coumaric acid CoA-ligase (4CL). Tyrosine, with 2-oxoglutarate as a co-substrate, is transaminated by the pyridoxalphophate-dependent tyrosine aminotransferase (TAT) to 4-hydroxyphenylpyruvic acid (pHPP). Hydroxyphenylpyruvate reductase (HPPR) is the enzyme responsible for the NAD(P)H-dependent reduction of pHPP. This enzyme, considered to be the first specific key enzyme responsible for the biosynthesis of RA, was first characterized in cell-free extracts obtained from suspension-cultured cells of *Coleus blumei* [71,72] and was then purified and sequenced [73]. 

The two intermediary precursors (pHPL and 4-coumaroyl-CoA) are coupled by ester formation to 4-coumaroyl-4’-hydroxyphenyllactic acid (4C-pHPL), with release of coenzyme A. The condensation reaction is catalysed by 4-coumaroyl-CoA:4’-hydroxyphenyllactic (CHPL) acid 4-coumaroyltransferase from the BAHD acyltransferase family, commonly referred to as rosmarinic acid synthase (RAS). RAS transfers the 4-coumaroyl moiety to the aliphatic OH-group of pHPL (Figure 2). Two meta-hydroxylations of the 4-coumaroyl moiety in the ester by two distinct (3- and 3′-) cytochrome P450 monooxygenases from the CYP98A family convert CHPL to RA (Figure 2). 

Only four of the enzymatic activities involved in this biosynthetic pathway seem to be specific to RA biosynthesis [74]. The enzymes PAL, C4H, and 4CL belong to the general phenylpropanoid pathway and are highly prevalent in land plants, as they catalyze the precursors for the formation of lignin and other phenolic compounds. TAT is also considered as a primary enzyme because it forms pHPP, which is needed for the biosynthesis of tocopherols and plastoquinones [75]. 

The conversion of hydroxypyruvate to glycerate by either a NADH-dependent peroxisomal hydroxypyruvate reductase (HPR) or a cytosolic NADPH-dependent HPR2 during photorespiration [76] resembles the stereospecific reduction of pHPP by HPPR. In fact, HPR2 from *Arabidopsis thaliana* heterologously expressed in *E. coli* accepted hydroxyphenylpyruvate as a substrate, even though *A. thaliana* does not biosynthesize RA [66]. It is currently under investigation whether HPRR is related to cytosolic HPR and if it should be regarded as a key enzyme in RA synthesis.

The enzymes involved in the last three steps of RA biosynthesis, RAS and the 3-and 3’-hydroxylases, have been characterized in cellular and subcellular preparations of suspension cells of *C. blumei* [77,78]. The high sequence similarity between hydroxycinnamoyl transferases and the meta-hydroxylases suggests they are closely related, but expression studies in heterologous systems have shown that the enzymes from *C. blumei* are specific for substrates involved in RA biosynthesis [79,80].

A list of all the RA biosynthetic enzymes whose full-length cDNA sequence has been described is included in Petersen [66]. The RAS gene has been cloned from *C. blumei*, *Melissa officinalis*, *Lavandula angustifolia*, and *Salvia miltiorrhiza*, showing that it has a high homology with genes encoding hydroxycinnamoyl transferases. CYP98A4 has been cloned from *Lithospermun erythrorhizon* (CYP98A46), *Ocium basilicum* (CYP98A13), *C. blumei* (CYP98A414), and *S. miltiorrhiza*. 

The extensive literature available about the bioactivity of RA reflects it has been the subject of considerable study [81,82]. Due to its antioxidant, antimicrobial, anti-inflammatory, antimutagenic, antiviral, anti-allergic, and anticancer activities, RA is used to treat peptic ulcers, arthritis, cataracts, cancer, rheumatoid arthritis, and bronchial asthma, among other illnesses [65]. The biological activities of RA have led to another use, mainly in Japan, as a food preservative to extend the shelf life of fresh seafood [83]. The biological activities of RA are summarized in Table 2.

An outstanding property of RA is its antioxidant activity, which is based on its ability to stabilize membranes and prevent free radical movement, consequently protecting the membranes against oxidation [84]. This activity was confirmed when it was demonstrated that RA increases the general stability of the spherical vesicles known as liposomes [85]. At the same time, Vostálová et al. [86] showed that RA significantly reduced the generation of ROS and decreased the secretion of IL-6 from T cells and macrophages, thereby avoiding the UVB-induced formation of human keratinocytes. An important application of RA as an antioxidant is that it significantly decreases the side effects involving DNA/chromosome damage caused by the anticancer compound, doxorubicin [87].

Alzheimer’s patients are known to have amyloid-β plaques in their brain, so there is considerable interest in finding a way of preventing the aggregation of these peptides. The use of orally administered RA has proved to be effective in inhibiting different pathways leading to the formation of these plaques [88]. Fallarini et al. [89] showed that low concentrations of RA exerted a protective effect on neurons against one of the most common neurodegenerative diseases, known as amyotrophic lateral sclerosis. Additionally, using mice as an animal model, Kim et al. [90] showed that RA inhibited angiogenesis on the retinal surface, by impeding the growth of retinal endothelial cells. In an in vitro angiogenesis test, RA also inhibited the formation of tube-like structures characteristic of this pathological vascularization. 

RA is also reported to suppress the fibrosis process and improve the biochemical indicators and morphology of the pathological tissues in a rat model with liver fibrosis [91], attributed to a negative effect on liver cytokines as well as the expression levels of a related gene. An apoptotic effect of RA has been described by Hur et al. [92] and strong evidence presented for its anticancer activity, notably a metastasis inhibitory capacity, after long-term consumption of large amounts in the diet [93]. Recently, it has been demonstrated that RA-enriched methanolic extracts obtained from *S. khuzistanica* cell suspensions have an apoptotic effect on MCF-7 cells through activation of the extrinsic pathway by increasing caspase 8 activity [94].

## 3. Lamiaceae Members: A Rich Source of Phenolic Antioxidants

The Lamiaceae (the mint family), the largest of the plant families, comprises about 250 genera and more than 6.000 species, which are distributed throughout the world, with a particular concentration in the Mediterranean region [95]. The plant stems, which can be herbs, shrubs, or trees, are often square in cross-section, and contain iridoids and phenolic glycosides [59,95].

The Lamiaceae contain many economically important species, used for their essential oils or as spices or herbs, including *Ocimum* (basil), *Thymus* (thyme), *Origanum* (oregano), *Rosmarinus* (rosemary), *Mentha* (peppermint, spearmint), *Lavandula* (lavender), *Marrubium* (horehound), *Nepeta* (catnip), *Salvia* (sage), and *Satureja* (savory). They are known as a rich source of plant antioxidants, especially phenolic acids and flavonoids. 

### 3.1. Savory (Satureja L.)

The genus *Satureja* L. belongs to the subfamily Nepetoidae, and tribe Mentheae, and comprises approximately 200 species, which are mainly aromatic and range from herbaceous plants to shrubs, with a wide distribution in the Mediterranean area, Asia and America [96]. The plants grow in areas with humid climates and deep soils as well as in rocky areas with arid, sunny climates. Iran is one of the most important genetic resources of *Satureja* in the world, and fourteen wild species, *S. sahandica*, *S. edmondi*, *S. intermedia*, *S. khuzistanica*, *S. mutica*, *S. rechingeri*, *S. isophylla*, *S. atropatana*, *S. spicigera*, *S. bachtiarica*, *S. montana*, *S. macrantha*, *S. laxiflora*, and *S. hortensis*, grow in northern, northwestern, western, southwestern, and central parts of Iran [97,98,99]. 

Essential oils and extracts from *Satureja* species rich in biologically active compounds, like other plants in the mint family, are used today in the pharmaceutical and food industries [100,101]. The most important compounds in *Satureja* extracts are free phenolic acids such as caffeic acid derivatives, including RA and *p*-coumaric acid [101]. 

Among the different *Satureja* species, summer savory (*S. hortensis*) and winter savory (*S. montana*) are widely cultivated as vegetables and spice plants in many parts of Europe [102,103,104]. The perennial *S. montana* grows wild in Europe but not in Iran, whereas the annual *S. hortensis*, traditionally cultivated in home gardens in different parts of Iran, is also widely grown and produced in France, Hungary, and Spain. Usually planted in early spring, it flowers until late June, when it is harvested at the full flowering stage to extract the essential oil [105]. The dry vegetative herbage yield of *S. hortensis* is more than six tons per hectare and its aerial parts can be harvested several times a year. 

Production in agricultural systems requires cultivars with high quality bioactive compounds, high yields of essential oil and vegetative herbage, uniform germination, and resistance to biotic and abiotic environmental stresses. To date, three commercial cultivars (Saturn, Compacta, and Aromata) have been modified and registered in Poland and Germany [106]. In Iran, native accessions of this traditional crop are cultivated in different regions. However, the environmental conditions of the plant habitats have played an important role in the development of different chemotypes, which can lead to the gradual emergence of a specific chemical type with a genetic basis [107,108,109].

Carvacrol is the most important biologically active phenolic compound in the essential oil of the two cultivated *Satureja* species [100,101,110] and is responsible for their significant antioxidant activity [101,111]. These species are the richest sources of carvacrol in the Lamiaceae. In addition to peppermint, carvacrol has also been identified in plant species of the Chenopodiaceae, Plantaginaceae, Apiaceae, and Verbenaceae [110]. This phenolic compound also inhibits prostaglandin biosynthesis, which is an important mechanism in relieving pain and anti-inflammatory processes. Other reported biological activities of carvacrol are antispasmodic, inhibition of acetylcholinesterase, lipid peroxidation, free radical scavenging, and macrophage stimulation of white blood cells [112]. 

As mentioned above, free phenolic acids such as caffeic acid derivatives, including RA, have also been reported in *Satureja* species extracts. Some flavonoids, such as apigenin, luteolin, and cynaroside have also been detected in the plant extracts [113].

Among the different Iranian *Satureja* species, essential oil and extracts of *S. khuzistanica* are particularly rich in carvacrol and free phenolic acids, especially RA, and therefore have significant biological activity [114,115]. The presence of very high concentrations of phenolic compounds with medicinal effects makes this plant a valuable candidate for use in the pharmaceutical and food industries. In recent years, several medicinal preparations, such as Saturex and Dentol, have been formulated and marketed from this plant.

#### 3.1.1. *Satureja khuzistanica*: A Chemical Factory of Rosmarinic Acid

**Figure 3:**
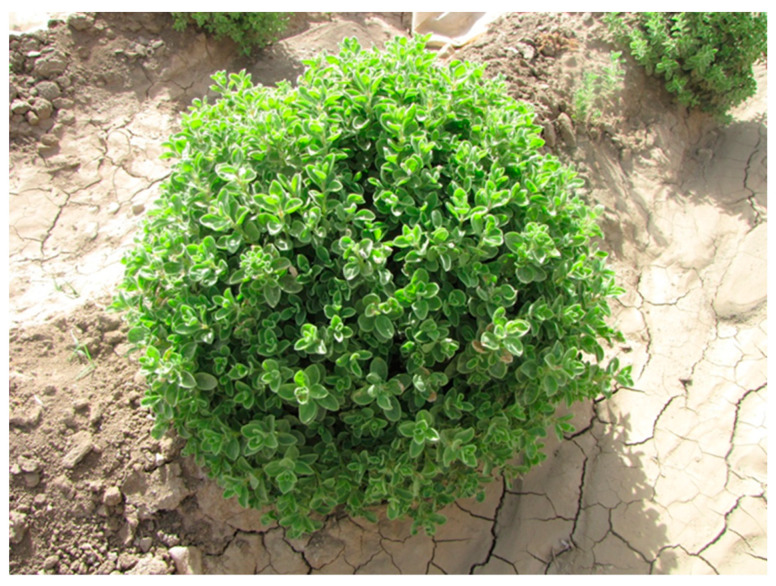
A cultivated *Satureja khuzistanica* Jamzad plant in southwestern Iran.

*S. khuzistanica*, which has the common Persian name of “marzeh khuzestani”, is endemic to western and southwestern areas of Iran, including the Lorestan and Kuzestan Provinces where it grows in dry, sunny limestone crevices [115]. A cultivated *S. khuzistanica* plant is shown in Figure 3. 

The plant, botanically characterized as a sub-shrub, has a branched shortly pubescent stem ±30 cm high, and is densely leafy. Flowering is from September to November [115,116].

*S. khuzistanica* has a strong fragrance and is a popular herb in parts of Iran where it is used by native populations as a herbal tea and in folk medicine for its analgesic and antiseptic properties. More recent biological activities reported for *S. khuzistanica* include antiviral, antibacterial, antifungal, antispasmodic and antidiarrhea or vasodilatory properties [117,118,119,120]. 

#### 3.1.2. Phytochemical Composition

Eighteen compounds have been identified in the essential oil of *S. khuzistanica* populations, representing 97.2–99.3% of the total oil composition [115]. The main component in all studied populations is carvacrol, found in a high percentage. Carvacrol is thought to play an important role in the adaptation of *S. khuzistanica* to harsh environmental conditions, such as a hot dry climate and calcareous, stony soils. The carvacrol precursors, *p*-cymene and γ-terpinene, have also been identified but in low concentrations. Carvacrol is a monoterpenoid phenol biosynthesized via the aromatization of γ-terpinene to *p*-cymene and then hydroxylated to *p*-cymene. This compound, with its two precursors, γ-terpinene and *p*-cymene, are the major components in several essential oils of the Lamiaceae family (e.g., in thyme, oregano, and savory oil). Carvacrol has a wide range of activities including anti-inflammatory, antioxidant, antimicrobial, and anticandidal properties [121,122].

The main components of *S. khuzistanica* essential oil in the studied populations are carvacrol (93.9%), eugenol (1.0%), p-cymene (0.8%), and thymol (0.6%) [123]. This suggests that the populations of *S. khuzistanica* are fundamentally homogeneous in their chemical composition. The essential oil constituents of other *Satureja* species have also been studied, revealing the existence of chemotypes in these species. For example, Sefidkon et al. [100] reported a chemical variation in *S. sahandica* oils from different populations and identified thymol (19.6–41.7%), *p*-cymene (32.5–54.9%), and γ-terpinene (1.0–12.8%) as the main oil constituents. Similarly, the essential oil composition of *S. montana* showed significant variations in the concentration of its major components, i.e., carvacrol (5.0–69.0%), linalool (1.0–62.0%), γ-terpinene (1.0–31.0%), and *p*-cymene (3.0–27.0%), indicating the existence of several chemotypes [109]. The variability of the essential oil composition of the cultivated accessions of *S. hortensis* has been recently reported [124] and carvacrol (42.0–83.3%), γ-terpinene (0.5–28.5%), and *p*-cymene (1.0–17.1%) were identified as the major components. 

The caffeic acid ester RA is known to have cognitive-enhancing effects and can slow down the development of Alzheimer’s disease. It also has cancer chemoprotection properties and anti-inflammatory, antibacterial, and antiviral activities [81]. The content of RA in MeOH extracts of *S. khuzistanica* samples varies significantly among different populations [115], in contrast with other phenols found in this plant species. Abdanan populations accumulate the highest levels of RA (1.81%), followed by those from Kaver (1.31%), while the lowest value has been obtained from Paalam populations (0.59%). Several authors suggest that RA is synthesized in response to stress produced under harsh environmental conditions or as a defense compound against plant pathogens [125,126]. In accordance with this theory, the higher RA levels found in the Abdanan and Kaver populations may be explained by the very hot and dry conditions where the plants grow.

## 4. Approaches to the Biotechnological Production of Rosmarinic Acid

The high demand for many medicinal plants has led to massive overharvesting and many of them have become endangered species in their original habitats. An important challenge for plant biotechnology is to find alternative sources of biologically active SMs. Biotechnological platforms based on plant cell cultures have been successfully developed for several medicinal plant species [30,82,127,128]. Among the methods used to improve the biotechnological production of SMs are screening and selection of highly productive cell lines, as well as the optimization of culture conditions and induction of secondary metabolism by the use of elicitors [54,55,128]. When undifferentiated cultures such as calli and cell suspensions are used to produce the target compounds, results are often poor.

**Figure 4:**
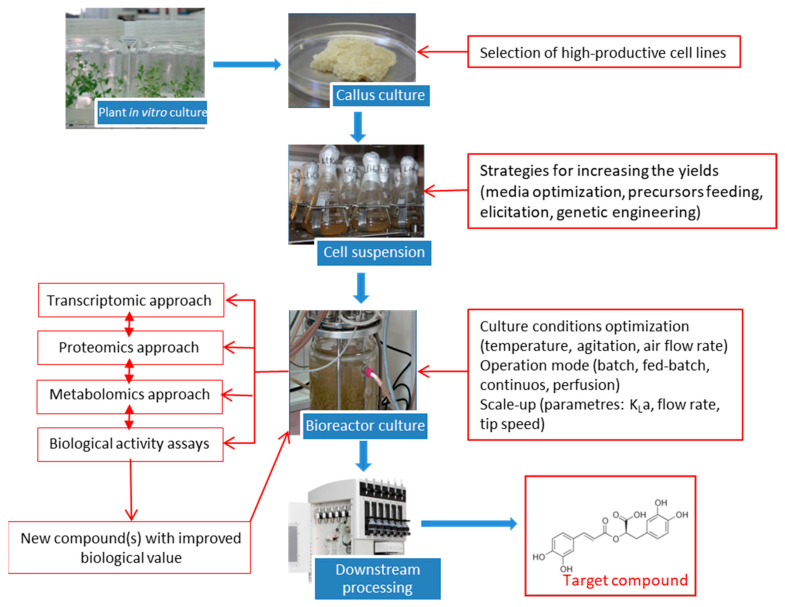
State-of-the-art biotechnological production of plant secondary metabolites in plant cell cultures (modified from Georgiev et al. [129]).

Currently, it is potentially possible to induce callus cultures from practically all plant species, although some species are more recalcitrant. Mineral nutrient composition, the type and concentration of plant growth regulators, as well as the source of explants are factors to be considered in callus induction (Figure 4). In vitro techniques can generate somaclonal variation: in this case, the selection of highly productive cell lines is another strategy for the successful production of plant SM [129]. As growth and secondary metabolism are often antagonistic processes due to competition for the same precursors, in biotechnological processes it is often necessary to change conditions optimal for growth to achieve high productivity of the target compound. The use of biosynthetic precursors and elicitors are widely used strategies for this purpose.

Another key issue in the development of a biotechnological process is scaling up the culture to bioreactor level. The main factors in this process include the selection of a suitable method for the process mode (batch, feed-batch, perfusion, etc.), and bioreactor type (stirred, airlift, bubble column, wave, etc.).

Recently, functional genomics (including transcriptomics and proteomics) synergically coupled with metabolomics have led to a systems biology approach, which potentially allows a full exploration of the biochemical machinery of plant cells and consequently their biosynthetic capacity can be more efficiently exploited [130]. In *Taxus media* cell cultures elicited with methyl jasmonate (MeJA), this approach recently enabled the isolation of 667 gene sequence tags (using cDNA-amplified fragment length polymorphism analysis (cDNA-AFLP) whose expression was modulated by the elicitor. A new gene was cloned from these tags and expressed in vitro and the results allowed the identification of Taximin, a new master regulator of taxane biosynthesis that could be used to improve the biotechnological production of taxol in *Taxus* sp. cell cultures [131]. Combining metabolomics analyses with biological assays (testing for anticancer, anti-inflammatory activities, etc.) will facilitate the discovery of new phytochemicals with improved therapeutic properties.

The current possibilities that plant biotechnology offers for SM production and the important pharmacological activities of RA have prompted many researchers to try to produce this compound in biotechnological platforms, such as shoots [132], cell suspensions [65], and hairy root cultures [133].

### 4.1. Plant Cell Cultures for RA Production

**Figure 5:**
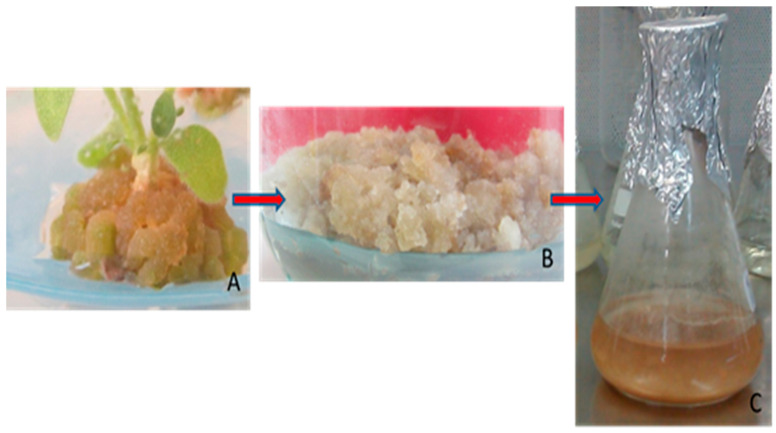
Steps for obtaining cell suspensions of *S. khuzistanica*. (**A**) Plantlet in vitro culture developing callus; (**B**) Callus culture; (**C**) Cell suspension.

Numerous species have been used to produce RA in plant in vitro cultures, such as *Anchusa officinalis*, *Eritrichium sericeum*, *Lithospermum erythrorhizon* (Boraginaceae), *Agastache rugosa*, *Coleus blumei*, *Hyssopus officinalis*, *Lavandula vera*, *Ocimum basilicum*, *O. sanctum*, *Salvia officinalis*, *S. chamelaeagnea*, *S. fruticosa*, *S. miltiorrhiza*, *S. maxima*, *S. verde*, *Zataria multiflora* (Lamiaceae), and *Anthoceros agrestis* (Anthocerotaceae), among other species [81], and more recently in *S. khuzistanica* [53]. The different stages in the development of RA-producing cell cultures of *S. khuzistanica* are represented in Figure 5. Biotechnological production of RA in plant cell cultures has achieved high yields, because this compound belongs to the so-called preformed secondary metabolites in plants, which are persistently biosynthesized [125].

The difficulties of RA production through field crops are being exacerbated by climate change and geo-political problems. In this context, green factories based on cell and organ cultures are emerging as an alternative bio-sustainable and eco-friendly source of high value bioactive plant secondary metabolites, including RA [134,135,136].

The first high RA production in cell suspension cultures was reported in sage (*S. officinalis*) by Hippolyte et al. [137]. The growth capacity and production of RA by these cells were modified by the culture conditions, leading to a 10-fold increase in RA production, and attaining 6.4 g/L under optimal conditions. Suspension cultures of *C. blumei* accumulated high amounts of RA in a medium with elevated sucrose concentrations [138]. Sucrose levels also affected RA production in cell suspension cultures of *Anthoceros agrestis* Paton: 2 and 4% sucrose were used, and cell suspensions achieved up to 5.1% dry weight (DW) of RA at day 8 in the medium supplemented with the lower concentration of sucrose [139]. In contrast, 7% sucrose was the optimal concentration for increasing RA production in cell cultures of *L. vera* [140]. Sugar-feeding experiments have also been carried out in callus cultures of *Zataria multiflora*, and the best source of sugar was 75 g glucose L^−1^ culture medium, when the callus achieved 158.26 mg RA g DW^−1^, a content 13-fold higher than the maximum reached in in vitro micropropagated shoot cultures [132].

Periodic culture perfusion increased cell density and RA production in *Achusa officinalis* cell cultures. In these conditions the maximum RA production was reached with an inoculum size of 4 g DW L^−1^ [141]. In order to increase RA levels, a perfused-batch culture was developed in shake flasks [142]. This strategy involved an intermittent medium exchange and the results showed a 2.3-fold increase in dry biomass and 2.2-fold rise in RA production in comparison with non-perfused cultures.

RA contents of callus cultures of *S. chamelaeagnea* induced on Murashige and Skoog culture medium containing 1-2 mg of 2,4-dichlorophenoxyacetic acid (2,4-D) were higher than the levels achieved by shoots induced on the same medium supplemented with 1 mg L^−1^ benzyladenine (BA) [143]. Biotechnological production of RA in callus, cell suspension, and root cultures of *S. fruticosa* was studied by Karam et al. [144]. The highest production was reached by 5-week-old calli (2.12 mg 100 mg DW^−1^), being 10-fold higher than in organs of field-grown plants.

Other reported sources of RA are calli and cell suspensions of *A. rugosa* and *E. sericeum*. After 10 days of culture, cell suspensions of *A. rugosa* reached the maximum growth and RA production when cultured in B5 liquid medium together with 2 mg L^−1^ 2,4-D and 0.1 mg L^−1^ of benzylaminopurine (BAP) [145]. *E. sericeum* root lines derived spontaneously from calli produced up to 4.50% DW of RA, whereas the original callus line achieved only 2.04% [146]. 

In plant cell cultures an elicitor can be defined as a factor that promotes the biosynthesis of secondary compounds with phytoalexinic activities. Classically, elicitors have been classified in two types, abiotic or biotic, according to their chemical nature and their exogenous or endogenous origin [147]. Several elicitors, such as yeast extract (YE), methyl jasmonate (MeJA), salicylic acid (SA), and *Pythium aphanidermatum* extracts, have been used to enhance the RA production in in vitro cultures, and their effects have been reviewed [83]. Mizukami et al. [148] achieved very good results, increasing RA production up to 10-fold, by eliciting cell suspensions of *L. erythrohizon* with 100 μM of MeJA. More recently, Khojasteh et al. [54] showed the positive effect of MeJA in cell cultures of *S. khuzistanica*, achieving a production of 245 mg g^−1^ DW after a week of elicitation. Abiotic elicitors such as vanadyl sulphate have been successfully applied to increase RA production in cell cultures of *Lavandula vera* MM [149]. Consequently, it is possible to infer that elicitation is an effective strategy for increasing the RA production in cell cultures, as reported for other secondary compounds. Some examples of the effect of elicitors on RA production are summarized in Table 3. These studies also show that there exists a positive relationship between RA production and the expression level of the main genes involved in its biosynthetic pathway.

Combining the effects of elicitation with feeding experiments has achieved successful results. The effects of different concentrations of sucrose, phenylalanine, and the elicitors YE and MeJA at several concentrations were tested with *Ocimum sanctum* cell suspension cultures. Production of RA reached a maximum when the culture medium was supplemented with sucrose at 5.0%, phenylalanine at 0.25 g L^−1^ and the elicitor MeJA [150]. In the case of *S. khuzistanica* cell cultures, 3.0 mM phenylalanine reduced the growth capacity of the cultures but enhanced RA production of cells, achieving a content of 227.76 mg RA g^−1^ DW at day 7 of culture [41].

Dimethyl sulfoxide (DMSO) is a permeabilizing agent that has been successfully used in cell and root cultures to promote the release of secondary metabolites from the cells to the culture medium. The excretion of target compounds to the culture medium facilitates the downstream processes to increase overall production. Cell suspensions of *C. blumei,* when maintained with the addition of 0.1% DMSO, presented a cell death rate lower than 15% in relation to the total cells, with a doubling time of 10.7 h and a RA production of 1.0–1.1 g L^−1^. This shows that this concentration of the permeabilizing agent is not harmful for the cells [151]. The response of the preconditioned cells (previously treated with 0.1% DMSO) was an improved cell growth and RA production when they were later treated with higher DMSO concentrations (0.5%). They reached a maximum of 2.85 g RA 100 g DW^−1^ in the culture medium, which represented 66.4% of the total RA produced. Immobilization is a plant cell culture technique that fixes the cells in a suitable matrix and prevents their movement into the culture medium. The first successful immobilization of plant cells was reported by Brodelius et al. [152], who entrapped *Catharathus roseus* and *Daucus carota* cells in alginate beads. Immobilization was proposed as a strategy to enhance the overall production of SMs in plant cell cultures. Immobilized cell cultures of *C. blumei* were developed to study the effect of the permeabilizing agent DMSO and growth regulators on biomass and RA production. Cells were immobilized in a support matrix composed of the fibrous skeleton of Luffa fruits. Immobilized conditions reduced the growth rate and RA production of cell cultures by half. The absence of growth regulators decreased the cell biomass and did not increase RA production. In this case, preconditioning treatment with 0.1% DMSO did not improve the cell adaptability to higher concentrations (0.5%) of the permeabilizing agent [151].

### 4.2. Biotechnological Production of RA at a Bioreactor Level

The last step in the development of a biotechnological process for producing phytochemicals is the scale-up from the laboratory to bioreactor level, while ensuring identical process characteristics [161]. Bioreactors were originally designed for the culture of microorganisms, which have different traits from plant cells and involve different processes compared to plant cell cultures (see Table 4). Plant cells have a diameter of 20–50 μm and a length of 100–500 μm, so are significantly larger than bacterial (<1 μm diameter) and fungal (5–10 μm diameter and <100 μm length) cells, with intracellular vacuoles that occupy up to 90% of cell volume. For this reason, they can be considered as “bags of water with thin cell walls” [23]. 

**Figure 6:**
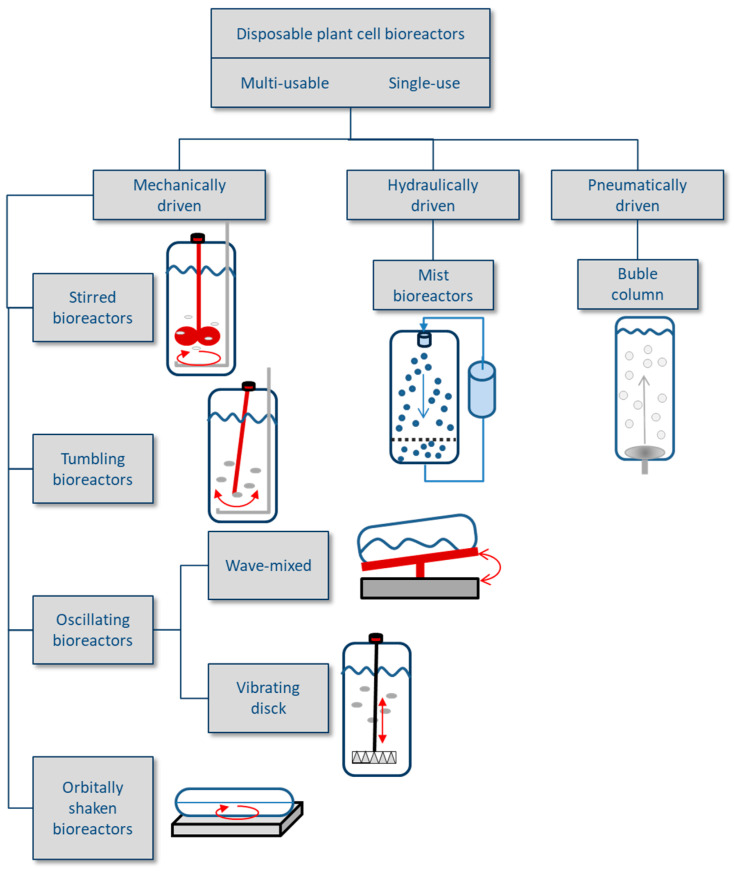
Disposable bioreactors classified according to their driving system (modified from Lehmann et al. [162]).

Plant cells are more sensitive to shear forces than microbial cells, and this fact conditions the stirring and aeration inside the bioreactor vessel [24]. The growth of plant cells is slower compared to microorganism cells and during the culture period they often release significant amounts of polysaccharides, increasing the viscosity of the culture medium. This may result in mass transfer limitations. Therefore, in many cases the design of the bioreactors has to be modified and adapted to the typical traits of the plant cell cultures in order to overcome these difficulties and implement the bioreactor systems at an industrial level (Figure 6). 

A wide variety of bioreactor designs have been used for the cultivation of plant cells. Traditional bioreactor configurations, such as stirred tank, bubble column or airlift bioreactors, have been utilized successfully for the production of phytochemicals in plant cell factories. Bioreactor design depends on whether the biosynthesis of the target compound is growth- or non-growth-associated and where the product is stored, either within the cell or secreted. If a plant secondary metabolite is produced during the exponential growth phase, only one reactor is normally required for growth and product recovery, but if the desired compound is synthesized after the cell growth phase, one reactor can be used during the exponential growth to increase cell biomass, and another one for metabolite production during the stationary phase, but currently also this is possible by using only a biorreactor by decoupling growth and production [24]. When the secondary compound accumulates inside the cell, the bioreactor is usually run-in batch or fed-batch (feeding) mode so that the cells can be permeabilized to release the product after the run is completed. If the product is secreted to the media, a continuous bioreactor can be used, and the compounds of interest can be removed as they are synthesized [24].

A bioreactor could be defined as a closed system where the producing organism synthesizes a target product, with guaranteed control of the process conditions. In a classical reusable bioreactor, the cultivation container is made of glass or stainless steel, but disposable bioreactors (Section 4.3) are being increasingly used [162]. Both kinds of bioreactors, reusable and disposable, have been successful applied for the production of plant secondary compounds in biotechnological systems [163].

The biotechnological production of RA has been carried out using different bioreactor types. *Anchusa officinalis* cell suspensions were cultivated in a stirred prototype bioreactor (2.3 L working volume) with an internal crossflow filter working as an automated perfusion device, where the cells were retained while fresh medium was and spent medium was removed. In this bioreactor a two-stage culture was successfully performed and after a culture period of 17 days the harvested biomass was 26 g DW L^−1^ and RA productivity 94 mg L^−1^ day, a productivity 3-fold higher than achieved in a batch mode [164]. The same authors subsequently developed a new two-stage perfusion culture with a high-density cell suspension. The best results were obtained when *A. officinalis* was cultivated in batch mode for 10 days in B5 medium supplemented with 3% sucrose and 0.25 mg L^−1^ NAA, followed by perfusing the culture with B5 medium with the sucrose concentration increased to 6 % at a constant perfusion rate of 0.1 day^−1^ [165]. 

In *Lavandula vera* MM cell suspensions, the effect of the temperature (T) on growth and RA production was investigated in a stirred bioreactor of 3 L, showing that a T lower than 26 °C was not suitable for biomass and RA production [166]. The best production was achieved when cells were cultivated at 30 °C. In the same system, the relationship between dissolved oxygen (DO) and the agitation rate was also investigated. After 12 days of culture, the cell suspension accumulated the highest amounts of biomass (34.8 g L^−1^) when 50% DO was supplied and the agitation rate was 100 rpm, whereas the highest RA production (1.8 g L^−1^) was achieved with 30% DO and an agitation speed of 300 rpm [167].

The systematic optimization of culture conditions for the biotechnological production of RA has been scarcely investigated. One exception is the work carried out by the Pavlov group [168], who developed and applied a polynomial regression model to describe the production of RA in a stirred 3 L tank, taking into account the DO concentration, agitation, and temperature. This was followed by a statistical optimization using a simple modified method. In optimized conditions, RA productivity was 3.5 g L^−1^, 2-fold higher than in the shake-flask stage, the optimal culture conditions being 50% air saturation, 400 rpm, and 29.9 °C.

Considerable progress has been made in the biotechnological production of RA, although much more work is still necessary. Key strategies for speeding up the process development and enhancing the biotechnological production of RA include the implementation of disposable bioreactors in fermentation procedures, the use of new elicitors and/or permeabilizing agents, as well as attempting to develop metabolic engineering approaches for the design of new cell lines with an improved capacity to biosynthesize/accumulate RA.

As mentioned above, despite the interest of RA and the extensive published work on its biotechnological production in plant cell and hairy root cultures, very few studies have been carried out at bioreactor level, and these studies have mostly been performed in reusable bioreactors. In recent years, reusable bioreactors have been replaced by disposable single-use bioreactors operating with a plastic bag or rigid plastic vessel. Currently plant cell cultures are being grown in disposable single-use bioreactors with volumes up to 400 L for the production of high-value compounds such as proteins and secondary metabolites [162]. These bioreactors have several advantages: they require no sterilization and cleaning, their usage is safe, time- as well as cost-saving, and economically friendly [169,170]. These features can be attributed to the plastic materials of the plant cell culture bags, which are made from multilayered, gamma-irradiated films in the majority [171]. There have been no reports of interactions between the culture media and the inner contact layer of the bags having any negative influence on plant cell growth until this date [172]. However, a disadvantage of these bioreactors is that a new bag has to be used for each bioprocess, which increases the operational costs.

However, some disposable bioreactors have cultivation containers that can be used more than once and are called disposable multi-usable bioreactors [173,174]. Compared to single-use containers, disposable multi-usable versions are more complex and more time-consuming to work with, but they are cheaper to purchase [162]. Both multi-usuable and single-use disposable bioreactor types have been used to grow plant cell suspension, hairy root, and embryogenic cultures [24,174,175].

There are three main classes of disposable bioreactors described in the litearure: (1) mechanically driven, (2) hydraulically driven and (3) pneumatically driven systems [162]. Mechanically driven disposable bioreactors are most often used due to their scalability and good investigation. Mixing in disposable bioreactors is performed by rotating and tumbling stirrers, vibrating perforated disks, rocking and rising platforms, or orbitally shaken platforms (Figure 6). 

In addition to the mechanically driven disposable bioreactor types, pneumatically driven bubble columns are used for plant cell suspension cultures. In the more simply designed disposable bubble columns, mass and heat transfer is achieved by direct sparging of air/gas into tall cultivation containers. The resulting bubbling causes mixing and fluid circulation of the culture medium. A modification of the bubble column is the airlift bioreactor, which has inner draft tubes to improve the mixing and aeration of the culture broth [174]. In the context of RA production, a 5-L disposable pre-sterilized plastic airlift bioreactor has been applied for the culture of *Ocium basilicum* shoot cultures [176]. 

Disposable mist bioreactor systems are based on a disposable bag in which a mesh matrix can immobilize cells and support biomass growth. Nutrient mist reactors are gas-phase reactors that periodically provide in vitro plants with small droplets (0.01–1 μm) of culture medium. These bioreactors are preferred for propagating plant organ cultures such as embryogenic or hairy root cultures. The use of such temporary immersion systems reduces tissue hyperhydricity. Vitrification has been classically associated with a complete or partial immersion of plant tissues in liquid medium [177]. 

Disposable wave-mixed bioreactors are composed of an inflated pre-sterilized plastic bag that forms a disposable cell cultivation chamber containing the culture medium and cells. The bag is fixed on an electrically driven rocking unit, whose movement induces a wave, which introduces bubble-free oxygen into the culture medium from the headspace of the bag, and the surface of the medium is continuously renewed. The wave movement sweeps up cells and prevents them from settling in the bioreactor. It also positively influences the mass transfer and reduces shear stress on cells, and thus supports cell growth and product formation [159]. A more homogeneous energy dissipation compared to stirred cell culture bioreactors was found.

Although the different wave-mixed bioreactor types are based on an identical working principle, they differ considerably in the culture bag design (bag material, scale and dimension, and the type of employed sensor probes and filters) and in the platform movement [178]. Wave-mixed bioreactors are currently the most favoured disposable bioreactors for many plant cell cultures grown at pilot scale up to 100 L working volume (this is sufficient for the manufacture of bioactive substances for cosmetics industry and also selected products for pharmaceutical applications. For example, Mibelle Biochemistry and Sederma have already developed and manufactured bioactive products for cosmetics, such as Phyto Cell Tec Argan, Solar, Vitis, Malus domestica, Alp Rose and RESISTEM. Greenovation also uses this type of bioreactors to produce different therapeutic proteins in *Physcomitrella patents* cell suspensions [162]. Nevertheless, wave-mixed bioreactors like BioWave^®^, Wave BioreactorTM, and BIOSTAT^®^ CultiBag RM have been successfully used for the culture of, among others, *Vitis vinifera*, *Malus domestica*, *Nicotiana tabacum*, and *Hordeum vulgare* cell suspensions as well as hairy root cultures of *Harpagophytum procumbens*, *Hyoscyamus muticus*, and *Panax ginseng* [179]. This type of bioreactor can therefore be considered as a good option for the cultivation of plant cell suspensions at medium scale.

Regarding *S. khuzistanica*, two attempts have been carried out for the biotechnological production of RA in cell cultures of this plant species. In the first one, a wave-mixed bioreactor with a 1L working volume was utilized for a culture period of 21 days, eliciting the cells with 100 μM MeJa. In these conditions, a biomass productivity of 18.7 g L^−1^ d^−1^ with a maximum RA production of 3.1 g L^−1^ was achieved, demonstrating the suitability of this biotechnological platform for the production of this plant antioxidant [54]. In a second attempt with the same cell line, a 2 L bag with a working volume of 1 L, shaken in a Khuner orbital shaker in the dark at 25 °C and 35–38 rpm, with a shaking diameter of 50 mm, was used. In this case, the elicitor treatment with 1 μM coronatine significantly increased the RA production capacity, achieving a specific production of 338.2 mg g DW^−1^ at day 16, which was 1.7 times higher than in control conditions (untreated cells).

Although the elicitation conditions and the age of the cell line were different, when comparing the biomass and RA production, both biotechnological systems, the wave-mixed and the orbitally shaken bag, showed that biotechnological platforms based on *S. khuzistanica* cell suspensions are effective systems for the biosustainable production of this antioxidant. In both cases the growth rate and maximum RA production was higher at bioreactor level than in shake flasks. 

### 4.3. Use of New Elicitors/Permeabilizing Agents

Several elicitor treatments have been used to improve the biotechnological production of RA (Table 2), including fungal elicitors, cuprum ions, silver ions, salicylic acid and MeJA. Permeabilizing agents like DMSO can also facilitate downstream processes [83]. In this context, cyclodextrins (CD), which are cyclic polymers of D-glucose linked by α-1,4-glycosidic bonds [177], have been tested in plant cell cultures for the production of bioactive SMs [180,181]. CD have attracted considerable interest recently, because they can act as really elicitors increasing the production of plant secondary metabolites and not only provoking the release of the target compounds to the culture medium. [182,183,184]. 

Coronatine (COR) is a pathogenic toxin produced by *Pseudomonas syringae* that has been tested as an elicitor in plant cell cultures of *T. media* and *T. globosa* [185,186]. It acts as a molecular stimulator of the isoleucine-conjugated form of jasmonic acid (JA-Ile), but being more stable, its mechanism of action is similar to that of the elicitor MeJA [187]. The addition of COR to cell cultures of *T. media* and *T. globosa* was more effective than MeJA in increasing taxane production, even at much lower concentrations. The taxane yields reached in *Taxus* spp. cell cultures treated with COR have been up to 5.3 times higher than those obtained with MeJA [185,186]. COR has also been successfully utilized to improve RA production in *S. khuzistanica* cell cultures. As COR significantly reduced the DW, the productivity of RA initially also decreased significantly after the elicitation. However, at day 21 (168 h after elicitation), the RA production levels of COR-treated cells started to overtake those of the control cells, reaching more than 2600 mg·L^−1^ after 240 h, which was significantly higher than the control (untreated cells) [94].

Thus, in most cases, the combined use of elicitors and permeabilizing agents can enhance SM biotechnological production [188] and could represent a suitable approach for increasing RA production in plant cell cultures of different plant species. It has been shown that elicitation of plant cell cultures with MeJA and CD enhances the accumulation of other phenolic compounds, such as resveratrol in *Vitis vinifera* [183] and silymarin in *Silybum marianum*. The production of other types of plant SMs has also been increased by this joint treatment, for example, aromadendrene in *Capsicum annuun* cell cultures [189], taraxasterol in *Solanum lycopersicum* [190], ajmalicine in *C. roseus* [191], and most recently taxol and related taxanes in *T. media* cell cultures [189]. In the same way, the joint application of COR or MeJA and CD dramatically increased the taxane production in *T. globosa* and *T. media* cell cultures [186,192]. These studies have demonstrated the effectiveness of this combined treatment and suggest it has the potential to improve the production of other SMs such as RA. Accordingly, Khojasteh et al. [54] demonstrated that in MeJA-elicited *S. khuzistanica cell* cultures treated with CDs only a small quantity of RA accumulated in the culture medium, and its cell content decreased significantly, probably due to the degradation of the bioactive compound by cellular apoplasts when released to the culture medium.

### 4.4. Metabolic Engineering Approaches

**Figure 7:**
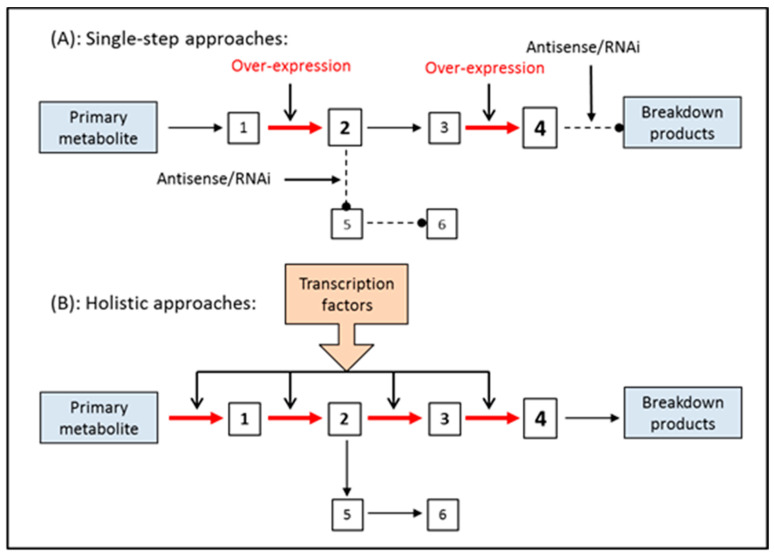
Comparative scheme of single-step and holistic approaches in plant metabolic engineering (modified from Onrubia et al. [187]).

Plant metabolic engineering offers a set of tools for overexpressing or silencing genes to modulate carbon flux in a biosynthetic pathway by targeting single steps. Alternatively, the expression of regulatory genes can be modified to establish multiple controls over one or more pathways in the cells (Figure 7) [187]. As previously mentioned, the biosynthetic pathway of RA is well elucidated, but its regulation remains unclear, which is probably why only single-step and not holistic approaches have been developed until now for improving RA biotechnological production by means of metabolic engineering tools. 

One challenge in plant metabolic engineering is to find enzymes that limit the precursor-flow in metabolic pathways and may therefore be suitable targets for engineering. In this regard, in vitro plant cultures can constitute an excellent platform for basic studies and to identify bottlenecks in a SM pathway. It has been demonstrated that in elicited cell suspensions and hairy root cultures, high RA production is correlated with a high transcript accumulation of genes encoding key enzymes involved in RA biosynthesis. 

An increase in PAL activity has been reported in elicited cells of *C. blumei* as well as in the activity of the specific RA biosynthetic enzyme RAS [153]. The limiting role of the PAL pathway was further confirmed by Kim et al. [193], who demonstrated that PAL, 4CL, and C4H were more active in *Agastache rugosa* cell cultures elicited with MeJA. 

Enhanced activities of PAL and TAT have been demonstrated in high RA-producing cell lines of *S. milthorriza* elicited with MeJA [194], showing that increasing the precursor of both committed pathways in RA biosynthesis could be a successful strategy to increase its biotechnological production. On the contrary, in YE-elicited hairy root cultures of *S. miltiorrhiza* TAT activity rose notably, whereas PAL activity decreased rapidly [195]. In the same system, HPPR gene expression was correlated with higher RA production for the first time [196]. This was subsequently confirmed by Zhang et al. [197], who found that a high RA production is more closely correlated with the activation of the enzymes involved in the tyrosine-derived pathway than with those related with the phenylpropanoid pathway. 

In this scenario, Xiao et al. [198] overexpressed some genes encoding key enzymes in the biosynthesis of RA, SmHPPR (hydroxyphenylpyruvate reductase), SmC4H (cinnamic acid 4-hydrolase), and tyrosine aminotransferase (TAT), in hairy root cultures of *Salvia miltiorrhiza* and observed higher RA accumulation in the transgenic lines. The highest levels of RA (992 mg L^−1^) were achieved when *hppr* and *tat* genes were co-overexpressed. These results were partially confirmed by Barberini et al. [70], who demonstrated that in cell suspension cultures of *S. officinalis* RA biosynthesis is highly correlated with the expression of the *hppr* gene, suggesting that this step could be a bottleneck for RA production in the cells and consequently a relevant target for new metabolic engineering approaches.

In hairy root cultures of *C. blumei*, overexpression of *hppr* and *ras* genes had controversial effects. Three hairy root lines harboring the *hppr* gene under the control of the 35S-promotor were obtained, two lines showing a higher accumulation of the *hppr* transcript than the control (untransformed) lines. High expression levels of the gene were correlated with an enhanced production of RA, which was up to 176% higher than in the control cultures. In contrast, expression of the *ras* gene in transformed roots carrying the *ras* overexpression construct did not increase and their RA production was generally lower than in the control root lines. The reduced expression of both genes in some of the hairy root clones obtained was attributed by the authors to co-suppression effects.

**Figure 8:**
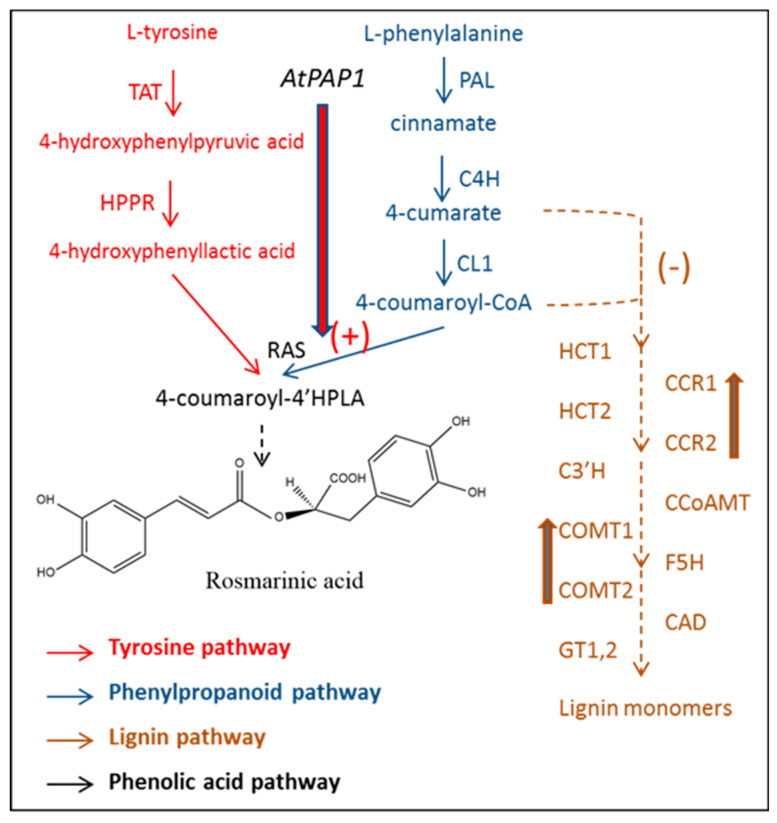
Methodological design based on the biotechnological strategy “increasing income and reducing expenditure” proposed by Zhang et al. [200] for improving RA production. CCR, cinnamoyl-CoA reductase; CCoAMT, caffeoyl-CoA O methyltransferase; C3′H, coumarate 3′-hydroxylase; C4H, cinnamate 4-hydroxylase; 4CL, hydroxycinnamate-CoA ligase; COMT, caffeic acid O-methyltransferase; GT, glycosyl transferase; HCT, hydroxycinnamoyl transferase; HPPR, hydroxyphenylpyruvate reductase; PAL, phenylalanine ammonia lyase; RAS, rosmarinic acid synthase; TAT, tyrosine aminotransferase.

Transgenic plants of *Perilla frutesces* overexpressing the tyrosine aminotransferase (TAT) gene were obtained by *Agrobacterium*-mediated transformation with the vector pCAMBIA23400-35S, which contains PfTAT under the control of the 35S promoter [199]. Transgenic plants accumulated higher contents of RA, which was correlated with the transcript level of the PfTAT gene. A new, very interesting metabolic engineering approach has been designed by the group of Zhang et al. [200] based on “increasing income and reducing expenditure”. To perform this, the Arabidopsis Production of an Anthocyanin Pigment transcription factor (AtPAP1) under the control of the 35S promoter was overexpressed in the high RA-producing plant species *S. miltorrizha*. AtPAP1 belongs to the MYB family of transcription factors and controls anthocyanin biosynthesis in plant tissues (Figure 8) [201]. 

Ectopic expression of AtPAP1 increases the accumulation of anthocyanins in *Arabidopsis* [202], tobacco [203], tomato [204], canola [91], and *Taraxacum* plants. Zhang’s group had previously demonstrated that overexpression of AtPAP1 in *S. milthorrizha* not only increased the production of RA up to two-fold, but also enhanced lignin accumulation [205]. As RA and lignins are in competition for the same precursors (Figure 8), in a later experiment, the same authors increased the biosynthesis of phenolic precursors by the ectopic expression of AtPAP1 and simultaneously decreased lignin biosynthesis via co-supression of two key enzymes involved in this process, cinnamoyl-CoA reductase (SmCCR) and caffeic acid O-methyltransferase (SmCOMT), using chimeric RNAi technology. The obtained transgenic plants accumulated significantly higher levels of RA than the control plants, showing the suitability of this strategy to increase phenolic compounds such as RA.

## 5. Conclusions

The growing demand for natural antioxidants has brought many plant species to the brink of extinction in their natural habitats. In this review, we have focused on the polyphenol rosmarinic acid, providing a summary of its biological activities and main plant sources, and covering the approaches to its biotechnological production reported to date. In conclusion, we would like to highlight that the high yields of rosmarinic acid achieved in *S. khuzistanica* cell cultures indicates they have promising application for scaling up in a bio-sustainable plant biofactory dedicated to the production of this powerful natural antioxidant.

## Figures and Tables

**Table 1:** The most important classes of phenolic compounds reported in plant materials.

Class	Compound	Natural Source
Phenolic acids	Hydroxycinnamic acids (α-cyano-4-hydroxycinnamic, caffeic, cichoric, cinnamic, chlorogenic, diferulic, coumaric, ferulic, sinapinic acids)	burdock, hawthorn, artichoke, pear, basil, thyme, oregano, apple, aloe, echinacea, strawberries, pineapple, coffee, sunflower, blueberries, oats, rice, orange, peanut
	Hydroxycinnamoyl esters derivatives (rosmarinic, caftaric, coutaric, and fertaric acids, verbascoside)	wide range of aromatic plants, especially mint family, mullein
	Hydroxybenzoic acids (salicylic and gallic acids)	olives, green pepper, berries
Flavonoids	Flavones (apigenin, luteolin, tangeritin, chrysin, 6-hydroxyflavone, orientin)	citrus, tangerine, celery, broccoli, green pepper, parsley, thyme, dandelion, perilla, carrots,
	Flavonols (quercetin, rutin, fisetin, galangin, kaempferol, myricetin, azaleatin)	wide variety of fruits and vegetables
	Flavanones (blumeatin, butin, eriodictyol, hesperetin, hesperidin, homoeriodictyol, isosakuranetin, naringenin, naringin, pinocembrin, poncirin, sterubin)	citrus, apple, cereal grains
	Flavanols (catechin, gallocatechin, epicatechin)	cocoa beans, grape seeds, coffee, tea leaves, apple, apricot
	Flavononols (taxifolin)	citrus, apple
	Isoflavonoids (genistein, daidzein)	red clover, soy beans, cereal grains
	Anthocyanins (glycosides of cyanidin, malvidin, delphinidin, pelargonidin, peonidin, and petunidin)	red vegetables and fruits, ornamental pants, black rice, and black soybean
	Anthocyanidins (capensinidin, cyanidin, delphinidin, europinidin, hirsutidin, pelargonidin, petunidin, cyanidin, malvidin)	grapes, blueberries, roses, purple cabbage, radishes, purple yams
	Coumarins (coumarin, scopoletin, aesculetin, umbelliferone, aesculetin, herniarin, psoralen, imperatorin)	tonka bean, vanilla grass, sweet woodruff, sweet grass, sweet-clover, cassia, cinnamon, mullein, strawberries, black currants, apricots, cherries
	Xanthones (mangostin and mangiferin)	mango, mangosteen
Quinones	Anthraquinones (emodin, rhein)	rhubarb
	Naphthoquinones (lawsone, lapachol, juglone)	henna, lapacho tree, walnut tree
Essential oils	thymol, carvacrol, eugenol, guaiacol, syringol	thyme, savory, oregano, clove
Stilbenes	resveratrol, piceatannol, pterostilbene, gnetol	grapes
Lignans	silybin, sesamol, pinoresinol, cordigol	milk thistle, sesame seeds, olive oil

**Table 2:** Biological activities of rosmarinic acid (RA) (modified from Bulgakov et al. [81]).

Biological Activity	Potential Usage
Antioxidant activity and membrane stabilization ^a^	Protection against chemically induced chromosome breakage and primary DNA damage
Increase of the physical and oxidative stability of liposomes ^a^	
Reduction of the frequency of micronuclei and the extent of DNA damage induced by doxorubicin ^a^	
Suppression of UVB-induced alterations to human keratinocytes ^a^	Skin protection against UVB light
Reduction of IFN-γ and IL-4 production by activated T cells ^b^	Skin protection against atopic dermatitis
Protection of neurons against insults ^a^	Rosmarinic acid is a promising neuroprotective compound of potential use at the nutritional/pharmaceutical interface
Cognitive-enhancing effect ^b^	
Prevention of the development of Alzheimer’s disease ^b^	
Attenuation of the degeneration of motor neurons and extension of the life span of model mice ^b^	
Anti-angiogenic activity against retinal neovascularization ^b^	Treatment of retinopathy
Inhibition of TNF-α-induced ROS generation and NF-κB activation and activation of TNF-α-induced apoptosis ^a^	Promising for cancer prevention and treatment of a variety of human cancers that are resistant to chemotherapy
The long-term exposure of animals to RA in the diet is sufficient for cancer chemoprevention ^b^	
Inhibition of bone metastasis from breast carcinomas ^b^	
Antifibrotic activity ^a,b^	Drug candidate for ameliorating liver fibrosis
Dramatic apoptotic activity on potentially pathogenic CD4^+^CD45RO^+^ effector T cells ^a^	Treatment of rheumatoid arthritis
Inhibition of caspase-1 activity, mitochondrial apoptotic pathway and activation of NF-κB by cisplatin ^a^	Prevention of harmful side effects of anticancer agents in patients undergoing chemotherapy

^a^ in vitro studies; ^b^ in vivo (animals); ^c^ in vivo (humans).

**Table 3:** Attempts to increase the biotechnological production of RA in cell suspensions by the use of elicitors and permeabilizing agents.

Plant Species	Elicitor Treatment	RA Production (% DW)	Reference
*Coleus blumei*	Fungal elicitor	2.1	[153]
	MeJA ^a^	3.3	[153]
	DMSO ^b^	2.9	[154]
*Eritrichium sericeum*	MeJA	5.3	[155]
*Lithospermun erythrorhizon*	MeJA	≈4	[156]
	YE ^c^	1.4	[148]
	Cuprum ions	1.5	[148]
*Ortosiphon aristatus*	YE	≈7	[157,158]
*Salvia miltiorriza*	SA ^d^	-	[159]
*Agastache rugosa*	YE	≈8	[160]
*Satureja khuzistanica*	MeJA	25	[54]

^a^ MeJA, methyl jasmonate; ^b^ DMSO, dimethyl sulfoxide; ^c^ YE, yeast extract; ^d^ SA, salicylic acid.

**Table 4:** The main differences between biotechnological processes based on microorganism and plant cell cultures.

Main Traits of the Culture	Microbial Cultures	Plant Cell Cultures
Size:	Small (1–10 µm)	Big (40–200 µm)
Growth form:	Single cells and clusters	Clusters and isolated cells
Growth rate:	Fast	Slow
Doubling time:	Hours	Days
Tolerance to shear stress:	Low	Moderate
Product accumulation:	Extracellular	Intracellular
**Main traits of the process**		
Culture medium composition:	Simple (few components)	Complex (Salts, sugars, PGRs ^a^, etc.)
Inoculum density:	Low	High (5–10%)
Temperature:	26–36 °C	25 °C
Aeration rate:	High	Low
Culture period:	Days	Weeks

^a^ PGRs, plant growth regulators.
